# Curious Tumour Attached to the Psoas Muscle

**Published:** 1831

**Authors:** 


### CURIOUS TUMOUR ATTACHED TO THE PSOAS MUSCLE. By Mr. WALDRON, Bath.

ON Nov. 12th, 1829, Mr. W. visited Lieut. H. æt. 38. He complained of constant pain and uneasiness referred to the back, and tenderness on pressure of the third lumbar vertebra; the aspect was sallow, the bowels irregular. Leeches and purgatives were prescribed. On the 15th, Mr. W., finding the patient could only lie on the left side with the knees bent, examined the abdomen and found a fulness in the course of the sigmoid flexure of the colon. He considered the case one of fæcal accumulations, and used means adapted to their removal, but without benefitting the symptoms. On the 17th the patient looked very ill, and Mr. W. again examined the abdomen, and thought he felt fluctuation in the fulness. The Lieut. said that he had suffered for several years from pain in the back and loins, that it had been growing worse for some months, and especially so within the last six or eight weeks. We need scarcely mention the treatment employed, as it did no good, and on the 24th Dr. Bowie met Mr. Waldron in consultation. On the 28th the consultants came to the conclusion that the case was one of psoas abscess, as the tumour dilated on coughing, and receded considerably when he lay upon his back. The patient sank rapidly and died on the 30th.

*Sectio Cadaveris.* "On the left side a fulness was visible, extending from the anterior and superior crista of the ilium, to the inner edge of Poupart's ligament, and there terminating with a slight bulge. Upon manual examination after death, a very indistinct fluctuation could be felt. On making an angular incision from the symphysis of the pubis, extending to the right and left ilium, a quantity of serous fluid escaped; on the left side, upon cutting through the parietes of the abdomen, and turning them back, the peritoneal covering of the left side was closely united, and could not be detached from a dark-coloured tumour which was now partially displayed to view. It appeared to occupy the greater part of the left side of the pelvis; from the close adhesions which had formed between this diseased mass, its peritoneal covering, the colon, rectum, and kidney of the left side, very considerable difficulty was experienced in its dissection, and by no means could it be satisfactorily effected. I first separated and secured by ligature, the large arch of the colon, above where the diseased structure commenced, in two places, and having then divided it, I proceeded to trace the sigmoid flexure downwards, but this I found impossible. The colon throughout, commencing from its sigmoid flexure, was embedded in an encysted tumour, of a dark colour, and when cut into and examined, evidently consisted of coagulated venous blood; the colon and rectum were so closely embedded in, and united to, this diseased mass, that they could not be separated from it; and appendiculæ pinguedinosæ throughout the whole course of the colon, even to some distance above the tumour, extending to its large arch, resembled so many black grapes, and when cut into, were found filled with dark-coloured venous blood. These bodies were found so closely adhering to the tumour, that it was impossible to separate them from it. The tumour was situated on, and adhering to, the psoas magnus muscle, extending to, and enveloping the left kidney, and appearing to take the course of the mesentery, filling up the loose fold which retains the sigmoid flexure of the colon, and passing downwards as far as the mesentery extends towards the rectum. It would appear that the mesentery thickened and condensed by chronic inflammation, formed a capsule for the tumour. The colon and rectum, when cut into and laid open, appeared healthy within. The whole of the intestines were highly vascular, and bore marks of inflammatory action, but more especially the large ones. The liver was of a light grey colour; the left kidney was very much diminished in size, and was surrounded by a yellowish substance, similar to pus. The spleen was of a light grey colour. A very considerable quantity of fluid was found in the abdomen, which, during the attempts made at dissecting out the tumour, became mixed with the coagulated blood, and added greatly to the difficulty in making a clear and satisfactory display of the parts, and rendered it impossible to discover the source of hæmorrhage."

We have seen the same sort of bloody tumour surrounding one kidney. It was like the contents of an aneurismal sac, consisting of dark coagulum in some places, yellowish lamellated coagulum in others. It was of great size, yet the vessel from which the bleeding had occurred eluded our detection. Of the symptoms during life we are ignorant. We suppose that if the tumour, in Mr. Waldron's case, had been of the hæmatoid character he would have recognized it as such.

